# The “FEEDS (FEeding Eating Deglutition Skills)” over Time Study in Cardiofaciocutaneous Syndrome

**DOI:** 10.3390/genes14071338

**Published:** 2023-06-25

**Authors:** Roberta Onesimo, Elisabetta Sforza, Valentina Giorgio, Germana Viscogliosi, Eliza Maria Kuczynska, Gaia Margiotta, Lucrezia Perri, Domenico Limongelli, Francesco Proli, Cristina De Rose, Donato Rigante, Antonella Cerchiari, Marco Tartaglia, Chiara Leoni, Giuseppe Zampino

**Affiliations:** 1 Center for Rare Diseases and Birth Defects, Department of Woman and Child Health and Public Health, Fondazione Policlinico Universitario A. Gemelli IRCCS, 00168 Rome, Italy; roberta.onesimo@policlinicogemelli.it (R.O.); valentina.giorgio@policlinicogemelli.it (V.G.); gevisco@gmail.com (G.V.); elizamaria.kuczynska@policlinicogemelli.it (E.M.K.); gaia.margiotta01@icatt.it (G.M.); lucrezia.perri@guest.policlinicogemelli.it (L.P.); domenico.limongelli01@icatt.it (D.L.); francesco.proli@guest.policlinicogemelli.it (F.P.); cristina.derose@policlinicogemelli.it (C.D.R.); donato.rigante@unicatt.it (D.R.); chiara.leoni@policlinicogemelli.it (C.L.); giuseppe.zampino@unicatt.it (G.Z.); 2 Dipartimento Scienze della Vita e Sanità Pubblica, Facoltà di Medicina e Chirurgia, Università Cattolica del S. Cuore, 00168 Rome, Italy; 3 Feeding and Swallowing Services Unit, Department of Neuroscience and Neurorehabilitation, Bambino Gesù Children’s Hospital IRCCS, 00168 Rome, Italy; antonella.cerchiari@opbg.net; 4 Molecular Genetics and Functional Genomics, Ospedale Pediatrico Bambino Gesù, IRCCS, 00146 Rome, Italy; marco.tartaglia@opbg.net

**Keywords:** cardiofaciocutaneous syndrome, dysphagia, genotype–phenotype correlation, personalised medicine, transition, RASopathies

## Abstract

Feeding, eating and deglutition difficulties are key concerns in patients with cardiofaciocutaneous syndrome (CFCS). This study intends to quantify the development of feeding skills from birth to adulthood in patients with CFCS. Twenty-seven patients (eight males; mean age: 16.7 ± 8.3 years; median age: 15 years, age range: 1.5–38 years) with molecularly confirmed clinical diagnosis of CFCS were prospectively recruited from the Rare Disease Unit, Paediatrics Department, Fondazione Policlinico Agostino Gemelli-IRCCS, Rome, Italy, over a one-year period. Pathogenic variants along with key information regarding oro-motor features were collected. Sialorrhea was quantified using the Drooling Quotient 5. Feeding abilities were screened using the Italian version of the Montreal Children’s Hospital Feeding Scale (I-MCH-FS). The oral sensory processing section of the Sensory Profile completed the assessment. Mild-to-profuse drooling was experienced by 25% of patients, and food taste selectivity was a constant during infancy (65%), with persistence even beyond adolescence. Nineteen percent of participants with long-term enteral feeding dependency had *BRAF*, *KRAS* and *MAP2K1* mutations. These findings document that mealtime challenges in CFCS do not remain restricted only to the paediatric age, and that supportive care until adulthood plays a key role.

## 1. Introduction

Cardiofaciocutaneous syndrome (CFCS; OMIM #115150) is a rare autosomal dominant disorder that belongs to a group of syndromes overall known as the RASopathies [1]. CFCS was first described by Reynolds et al. in 1986 [2]. Although neither its incidence nor its overall prevalence are yet known to date, it was estimated to occur in 1:810,000 live births in Japan [3]. Diagnosis is suspected via clinical findings and confirmed via molecular testing [4,5]. CFCS is caused by germline mutations in four genes that encode components of the Ras/mitogen-activated protein kinase (*MAPK*) pathway [6], a fundamental intracellular signalling network guiding diverse biological functions such as proliferation, differentiation, survival and senescence. Specifically, de novo pathogenic variants in the *BRAF* (~75%) [4,7,8], *MAP2K1* and *MAP2K2* (~25%) [4,7] and *KRAS* genes (˂2%) [4,8] have been recognised as being causative of CFCS [4,8]. More recently, a pathogenic variant has also been reported in the *YWHAZ* gene [9]. All pathogenic variants promote the functional upregulation of these signal transducers, resulting in enhanced signalling through the MAPK cascade [10]. 

As far as the phenotype is concerned, CFCS partly overlaps with both Costello syndrome (CS; OMIM #218040) and Noonan syndrome (NS; OMIM #163950). CFCS-affected children present with reduced postnatal growth, failure to thrive, distinctive craniofacial appearance and most notably relative macrocephaly, a high cranial vault, usually curly hair, bitemporal narrowing and hypoplastic supraorbital ridge [4]. The clinical phenotype of CFCS is also characterised by congenital cardiac defects (i.e., pulmonary valve stenosis and hypertrophic cardiomyopathy, mostly associated with *BRAF* variants) [11], short stature, developmental delay/intellectual disability, gastrointestinal dysfunction, ectodermal abnormalities and musculoskeletal problems (e.g., reduced bone mineral density, kyphoscoliosis, pectus anomalies and generalised muscle hypotrophy) [4,12]. The most frequent structural anomalies of the central nervous system comprise ventriculomegaly, hydrocephalus and cortical atrophy [13,14]. Neurologic features of variable degrees are constantly present [15,16,17,18,19], including speech delay and/or marked learning disability, hypotonia at birth and motor delay [20]. Epilepsy, often requiring multiple lines of treatment, is also commonly reported in individuals with CFCS [16]. Intraoral findings include high-arched palate, anterior open bite and posterior crossbite [21]. Genotype–phenotype correlations have been reported [13].

Feeding difficulties and poor child growth are some of the first issues that a caregiver faces when coping with a CFCS diagnosis. Hypersensitivity of the oral cavity complicates the introduction of solid foods. Masticatory deficit is also very frequent. During infancy, failure to thrive is widely prevalent in this disorder [10]. From a gastrointestinal standpoint, gastroesophageal reflux disease (GERD), recurrent vomiting, nausea and aerophagia have been reported in infant and adult CFCS patients, and in the most severe cases, they lead to the necessity of artificial enteral nutrition via either nasogastric tube or, more rarely, gastrostomy. Intestinal dysmotility-related constipation can occur during infancy and last a lifetime, regardless of the genotype [14]. 

Recently, feeding disorders have gained a great deal of attention in RASopathies. However, their evolution from birth to adulthood has not been characterised in detail. With the aim of overcoming this lack of knowledge, we focused on the temporal trajectory of feeding abilities in CFCS.

## 2. Methods 

The Strengthening the Reporting of Observational Studies in Epidemiology (STROBE) guidelines for cross-sectional studies were followed (Supplementary Table S1).

### 2.1. Participants

We consecutively identified 33 unrelated individuals with a molecularly confirmed clinical diagnosis of CFCS. All participants were recruited among those routinely monitored at the Rare Disease Unit of the Paediatrics Department, Fondazione Policlinico Agostino Gemelli-IRCCS, Rome, Italy, from June 2021 to June 2022. The Local Ethical Committee approved the study as part of a large protocol evaluation on disability in rare disease patients. 

Participants were categorised in four main groups for further exploratory analysis: early childhood = 3–5 years; middle childhood = 6–11 years; adolescence = 12–21; adults > 21 years. 

### 2.2. Measures and Procedures

All subjects included in the study underwent a comprehensive paediatric clinical interview and examination. The feeding and swallowing assessment was conducted in accordance with the World Health Organization’s (WHO) International Classification of Functioning, Disability and Health (ICF) framework. Data on the retrospective assessment of feeding abilities from the neonatal age were collected for each participant. 

Sialorrhea was evaluated and quantified using the Drooling Quotient 5 (DQ5) [22], a semiquantitative, direct observational method that evaluates drooling by measuring leaked saliva from the mouth (so-called anterior drooling) [23]. Specifically, over 5 min, for every interval of 15 s, the presence or absence of drooling was determined [22].

The presence of feeding disorders was first screened thorough the administration of the Italian version of the Montreal Children’s Hospital Feeding Scale (I-MCH-FS) [24]. The scale is composed of 14 items and covers 7 feeding domains including oral sensory and motor abilities, mealtime behaviours, appetite, caregiver concerns about feeding, family strategies and reactions to their child’s feeding during mealtime [25]. 

Supplementary searches on oral sensory processing and feeding behaviour included the administration of the Sensory Profile (oral sensory processing section). The Sensory Profile (SP) [26,27] is a well-validated 125 proxy-reported questionnaire that uses a 5-point Likert scale (never = 5; seldom = 4; occasionally = 3; frequently = 2 and always = 1) to assess the child’s responses to daily sensory stimuli. The oral sensory processing section within the sensory processing category is composed of 12 items (#54–65), evaluating the type of neurological threshold (low or high) for oral stimuli. For the present study, the child version of the SP (3–10 years) was always preferred over the adolescent/adult one (>10 years) due to the patients’ cognitive impairments.

I-MCH-FS and the SP were administered to children in the early- (3–5 years) and middle-childhood age groups (6–11 years). Children with total enteral nutrition were excluded. 

Questionnaires were administered on the same day as the paediatric clinical assessment. The responders were the primary caregivers. Descriptive statistics were performed on demographic and clinical characteristics in the data set. Results are presented as mean ± standard deviation, median, interquartile range or percentage. The Kolmogorov–Smirnov test was performed to determine the normality of the patient’s data distribution. Data analysis was performed using GraphPad Prism version 9.1.2.

## 3. Results 

### 3.1. Participants 

Twenty-six patients (78%) agreed to be part of this study (eight males; mean age 16.7 ± 8.3 years; median age 15 years; age range 1.5–38 years). The Kolmogorov–Smirnov test revealed a normal distribution (D = 0.114, *p* = 0.84). The four main groups were composed of three patients in early childhood, seven in middle childhood, seven in adolescence and nine in adulthood. The multigene panel/sequential gene or more comprehensive genetic testing, when available, detected *BRAF* mutation in 21 patients, *MAP2K1* in 3 patients, *MAP2K2* in 1 patient and *KRAS* in 1 patient (Table 1). 

### 3.2. The Evolution of Feeding Abilities over Time

#### 3.2.1. Prenatal and Neonatal Period

A pathological increase in amniotic fluid volume was detected in 42% (*n* = 11/26) of pregnancies. Preterm birth affected 46% of infants (*n* = 12/26), mostly accompanied by floppiness. According to parents’ reports, a poor suckling ability was appreciated during the first period of life in 24 newborns, with half of them (*n* = 12) requiring total enteral nutrition (TEN), specifically through a nasogastric tube (NGT) (Table 2). Among the rare birth defects, intestinal malrotation was detected in one patient. 

#### 3.2.2. From 1 Month to 6 Months of Life

The introduction of semisolid food with a spoon was performed in 69% of infants (*n* = 18/26) within six months of age, with major difficulties in the acceptance of the proposed foods. Specifically, up to 30% of caregivers reported that their children showed food aversion for pureed foods and had an extended meal duration. 

At this age, 19% of infants (*n* = 5/26) were still tube-feeding-dependent. The remaining three children were exclusively bottle-fed for an extended time, specifically until the ages of 1, 2.5 and 9 years. For them, the introduction of semisolid food via a spoon and soft solid foods was delayed due to prolonged nonacceptance of consistencies other than semiliquid.

Overall, mild-to-severe food aversion was present in 65% of the infants (*n* = 17/26). 

#### 3.2.3. From 6 Months to the 1st Year of Life

Even after the first sixth months of life, 19% of infants (*n* = 5/26) with tube feeding dependence were not able to eat by mouth.

Among the weaned children, only half of them (*n* = 8/16) were able to chew solid foods upon reaching the age of one year. At this age, 57% of the whole cohort (*n* = 15/26) suffered from GERD, accompanied by recurrent vomiting episodes in four cases. 

#### 3.2.4. Over the 1st Year of Life

All of the five tube-fed children needed artificial enteral feeding support over a long period of time.

One patient was still tube-fed at 16 years old.

Full oral feeding was achieved in the remaining four tube-fed patients upon reaching the ages of 20 months, 2.5 years and 5 years, respectively (Table 2).

CFCS participants with a persistent worse outcome (long-term enteral-feeding-dependent) had *BRAF, KRAS* and *MAPK1* mutations. Participants requiring artificial enteral nutrition (any kind of device) had *BRAF* (*n* = 7/21), *MAP2K1* (*n* = 2/3), *MAP2K2* (*n* = 1/1) and *KRAS* (*n* = 1/1) variants. 

Despite growth, approximately one third of participants (*n* = 8/26) never reached the ability to appropriately chew solid foods. For them, immaturity of the chewing pattern did not evolve into more precise, stable, rotator movements of the oral muscle. Foods of room temperature were better tolerated than either hot or cold ones in a quarter of cases (*n* = 7/26), despite a tendency to prefer tasty foods. Scarce or even absent oral hygiene during childhood caused by low tolerance for a toothbrush into the oral cavity was seen in 42% of cases (*n* = 11/26).

#### 3.2.5. Adulthood

In terms of swallowing abilities, two out of the nine adult participants showed signs of oropharyngeal dysphagia. One of them (carrying a *MAP2K1* mutation), after removing enteral nutrition at the age of 2.5 years and starting to eat everything by mouth, required G-tube repositioning at the age of 36 years for partial nutritive support. This patient presented with diffuse cerebral atrophy and drug-resistant epilepsy.

In terms of food aversion, for most adults with CFCS, caregivers reported a mild-to-moderate improvement in food acceptance, although the permanence of food taste selectivity. In one case (*MAP2K1* mutation), severe food aversion persisted. 

### 3.3. Scale and Questionnaire

#### 3.3.1. DQ

Mild-to-profuse drooling was observed in a quarter of cases (*n =* 7/26). Constant drooling was a commonly complained-about issue by 11% of parents (*n* = 3/26), with DQ5 values of more than 18, while occasional drooling was experienced by 15% of patients (*n* = 4/26).

#### 3.3.2. I-MCH-FS and SP

Prior to the administration of scales, the clinical evaluation of the swallowing function showed that two cases were not able to safely swallow solid food, with indirect signs of bolus aspiration, namely cough, regurgitation vomiting or even choking (in one case), confirmed via videofluoroscopic swallowing study (VFSS). 

Responders were always the primary caregivers, specifically the patients’ parents. 

Out of the whole cohort, 42% of caregivers reported their child, regardless of age or gender, to always feel hungry (*n =* 11/26), even when often accompanied by severe food taste and consistency selectivity. 

I-MCH-FS and SP were administered to nine caregivers (mean child age = 7.7 years). For the I-MCH-FS assessment, the mean total converted score was 60.3 (range: 35 to 92). The majority of responders (*n* = 6/9, 66%) demonstrated mild-to-severe difficulties (mild: *n* = 4; severe *n* = 2). On average, mealtimes were considered to be quite difficult, characterised by a tendency to refuse to eat, mean 40 to 50 min to complete a meal and a tendency to gag or spit foods with consequent parental concern (Table 3).

For the SP, the total score fell within the ‘typical performance’ category for 55% of participants (*n* = 5/9), and the remaining 45% fell within the ‘probable and difference’ category. 

### 3.4. Other Findings

The comprehensive paediatric clinical interview and examination revealed the presence of cardiovascular defects in 20 participants, including pulmonary valve stenosis at birth in 23% of cases (*n* = 6/26) and mild dysplasia of the mitral valve in 30% (*n* = 8/26).

Magnetic resonance of the brain evidenced anomalies in 61% of participants (*n* = 16/26), including microcephaly associated with agenesis of the corpus callosum (*n* = 1/26), temporal cortical dysplasia (*n* = 1/26), mesial temporal hippocampal sclerosis (*n* = 1/26), regressive atrophic parenchymal manifestations (*n* = 1/26) and diffuse cerebral atrophy (*n* = 1/26).

With regard to age sub-groups, two out of the three participants in the 3–5 years age range underwent drug treatment for GERD, with 57% (*n* = 4/7) in the 6–11 years group and 28% (*n* = 2/7) in the 12–21 years group, while this was not observed for subjects with age > 21 years *n* = 0/9). One 3-year-old child underwent Nissen fundoplication surgery.

Seizures affected 46% of our cohort (*n* = 12/26), including generalised tonic-clonic, absence, complex partial seizures and infantile spasms. Developmental milestones were delayed in the majority of cases. Only two children reached the ability to walk in a timely manner, and 42% of the participants (*n* = 11/26) were able to walk alone within the first three years of life. The mean age of reaching this milestone was 4 years. Three adult participants needed support with walking. Neurocognitive evaluation evidenced a mild-to-severe cognitive impairment in all of the participants and autistic spectrum features in one case (*BRAF* mutation). Language abilities ranged from limited nonverbal communication to capacity to speak in full sentences. On average, children with CFCS spoke their first word at around 2 years of age, although one child remained nonverbal.

## 4. Discussion

Feeding and swallowing issues are highly common in patients with CFCS, often causing troublesome symptoms throughout their life. In this context, knowledge about the severity and evolution over time of the feeding difficulties encountered by these individuals enriches the description of the natural history of CFCS, helping clinicians to define the most appropriate follow-up and treatment strategies.

In the present cohort, feeding difficulties were a constant finding since before birth, as demonstrated by the high prevalence of reported polyhydramnios, namely a pathological increase in amniotic fluid, mainly due to disturbed foetal swallowing [28], as was described by Allanson et al. [14]. Soon after birth, a high prevalence of scarce sucking ability leading to a need for enteral nutrition support has been noted in almost all published cohorts, analogous to what was also observed for most infants with CS [29,30,31,32]. Despite these common aspects shared by both CS and CFCS, CS children face an improvement in feeding abilities during growth [33], requiring the placement of a gastrostomy tube that is usually removed at 4 or 5 years of life [29]. Such a finding is in contrast with what is observed in CFCS children who face persistent food aversion and oral hypersensitivity, therefore requiring enteral nutrition support even after early childhood [25]. An improvement in the early feeding difficulties was also reported in individuals with NS, with the major improvements reported after the second year of life [34]. Our study, about the temporal trajectory of nutritional issues, confirms how feeding difficulties in CFCS remain severe for an extended period of time, with a mean age for tube feeding removal of 7 years. In our study, food aversion, taste and food temperature selectiveness and scarce tolerance to the introduction of spoons or toothbrushes were observed. These findings further prove the high level of severity of feeding difficulties in this specific condition. Of note, our study highlights the failure to achieve the age-appropriate chewing pattern in children with CFCS, specifically in 30% of the orally fed children. 

The genotype–phenotype correlation between CFCS genes and feeding difficulties has been recently analysed [29], showing a relatively homogeneous and high prevalence of artificial enteral nutrition (with any kind of device), independent from the causative gene mutation (48–54%, *BRAF*; 40–67%, *MAP2K1*; 40–50%, *MAP2K2*) [14,15]. The prevalence of artificial enteral nutrition in our cohort is similar to the ones that have been previously reported in the literature. In addition, long-lasting feeding difficulties were experienced by 19% of participants in the studied cohort (with *BRAF, KRAS* and *MAPK1* mutations). CFCS-associated mutations affecting the *BRAF*, *MAP2K1*, *MAP2K2* and *KRAS* genes are predicted to cause an enhanced activation of signalling through the RAS-MAPK pathway [4]. While the collected data do not provide evidence for the occurrence of clear-cut genotype–phenotype correlations involving feeding difficulties, a larger series of affected subjects is required to more accurately explore this relevant clinical aspect.

As per the emerging need to address health challenges that patients need to face during growth and the lack of specific data in the literature, we also reported findings on adult individuals with CFCS. Our results suggest that older patients may face a worsening in their swallowing abilities over the years, with the consequent need for enteral nutrition support. Moreover, despite older age, food aversion may persist. These aspects, particularly if associated with severe epileptic encephalopathy and/or progressive neurological deterioration, which are commonly described in individuals with CFCS [3,13,25], may contribute to a persistence in the frailty of the patients with this condition from infancy to adulthood.

Although only reported by Armour et al. [15], drooling is a further sign of oral–sensory–motor difficulties experienced in CFCS, and it was also observed in 25% of our patients. Drooling has actually been often recognised as a health issue in children with disabilities, including cerebral palsy (CP) and other congenital syndromes [35], with all of its consequent psychosocial complications [36]. Drooling increases the burden of care for parents and their families and results in social isolation and associated low self-esteem. Current management options for drooling include conservative treatments (i.e., speech or behavioural therapy) and pharmacological therapies, such as the administration of anticholinergic drugs or of intraglandular botulinum neurotoxin type A (BoNT-A). Unfortunately, the effect of these medications is only temporary, with a median duration of 22 weeks for BoNT-A injections [37]. If patients are refractory or ineligible for conservative options, and long-term results are needed, surgical strategies, including duct ligation and submandibular or parotid gland excision, may be the option of choice [38].

Another interesting finding observed in the present study was the high prevalence (40%) of constant hunger, regardless of age or gender, which was remarkably reported to commonly occur in association with food refusal. Moreover, the administration of the I-MCH-FS allowed us to quantify feeding difficulties in the CFCS children, showing, in most cases, results above the normality range [24,25], confirming the extent of these issues experienced by the entire families and/or caregivers [13]. The described negative mealtime behaviours, including long feeding time and tendency to spit foods [29], are triggered by impaired oral sensory regulation, quantified in this study by the SP questionnaire. In addition to RASopathies [39], atypical sensory modulation has also been reported in other conditions, namely Smith–Magenis, Williams and X-fragile syndromes [40,41,42]. Tactile defensiveness aptitude has been confirmed to be strictly related to the behavioural challenges described for CFCS [40]. Feeding challenges may also be worsened by traits of autism spectrum disorders (ASDs), associated with developmental RAS/MAPK pathway dysregulation [43,44]. The literature has demonstrated how ASD children experience increased food selectivity, along with mealtime-related behavioural problems that further exacerbate caregivers’ distress. In this context, a combination of medical and behavioural intervention in highly structured settings has been demonstrated to be supportive for feeding issues in children with ASD [45].

The high prevalence of GERD occurring early in life reported in the studied cohort confirms previous findings [15,45,46]. GERD can cause CFCS children to associate feeding with pain, acting as a contributing factor to feeding aversion and failure to thrive [47,48]. Specifically, GERD can undermine the development of adaptive skills for feeding. Children may exhibit coughing, gagging and feeding resistance. Consequently, mother–child interactions resultingly become disturbed, and feeding becomes unpleasant and a cause of potentially aversive behaviours [49]. 

In conclusion, feeding issues are not only high during infancy and childhood in individuals with CFCS, but may persist into adulthood.

Therefore, it is of the utmost importance to plan a personalised program of care for these patients as early as possible in order to overcome their difficulties and to avoid further deterioration during adulthood. 

RASopathies, like other chronic disorders, present with a chronic and often life-threatening course [50]. The present findings indicate that the management of feeding issues in patients cannot be terminated when reaching adulthood, but should continue over the entire life-span.

## 5. Conclusions

Improving the diagnosis and management of rare diseases is a key current public health concern. Our data increase the existing knowledge on feeding and swallowing skills in those with CFCS over time, helping the comprehension of the natural history of this disorder. Understanding the feeding and nutritional challenges experienced in the transition period from youth to adulthood is also critical to ensure appropriate health interventions.

## 6. Limitations and Future Research

Some limitations should be noted. Despite covering a rather large cohort of cases, in consideration of the prevalence of this rare condition, the generalisability of these results should be validated by further extensive research. Due to the relatively small cohort size, we could not explore the occurrence of genotype–phenotype correlations. The assessment of possible patient stratification in the context of feeding difficulties requires a larger cohort size, eventually in the framework of a multicentric collaborative effort. Moreover, due to the presence of cognitive impairment, self-completion of the proposed scale and questionnaires was not achievable. The strength of this study is the use of well-validated scales and questionnaires, specifically translated and adapted for the Italian-speaking population. Future multicentre studies are needed to record the trend over time of feeding abilities in people with further chronic congenital disabilities. 

## Figures and Tables

**Table 1:** Details of our cohort of patients with cardiofaciocutaneous syndrome.

Participants (*n* = 26)	
Demographics at the Time of Our Study	
Age range (years)	1.5–38
Age groups *n* (%)	
3–5 years	3 (12)
6–11 years	7 (27)
12–21 years	7 (27)
>21 years	9 (34)
Mean age (years), SD age (years)	16.7 ± 8.3
Gender (M)	8
Genetics *n* (%)	
*BRAF*	21 (81)
*MAP2K1*	3 (12)
*MAP2K2*	1 (3.5)
*KRAS*	1 (3.5)
Features *n* (%)	
Seizures	12 (46)
**MR anomalies**	16 (61)

MR = magnetic resonance, SD = standard deviation.

**Table 2:** Genetic findings, long-term worst feeding outcome and main findings in newborns with cardiofaciocutaneous syndrome.

Pt.			Genetics		Findings					
	Age (y)	Gender	Gene	Variant	Polyhydramnios	Prematurity	Sucking Difficulty	Nasogastric Tube Feeding	Floppiness	G-Tube Timing (y)
#1	11	F	*BRAF*	p.Thr599Arg	+	+				
#2	14	F	*MAP2K1*	p.Pro124Leu	+	+	+	+		
#3	23	M	*BRAF*	p.Lys483Asn	+		+			
#4	30	F	*BRAF*	p.Gln257Arg						
#5	25	F	*BRAF*	p.Lys499Asn			+			
#6	3	F	*BRAF*	p.Gln257Arg		+	+		+	
#7	8	F	*BRAF*	p.Gln257Arg	+	+	+			
#8	18	F	*BRAF*	p.Trp531Cys	+	+	+		+	
#9	23	F	*MAP2K1*	p.Tyr130Cys	+		+		+	
#10	16	F	***BRAF***	**p.Asp678Glu**	+	+	+	+		**16**
#11	13	M	*BRAF*	p.Asp638Glu			+			
#12	7	F	*BRAF*	p.Asn581Asp			+			
#13	28	F	*BRAF*	p.Gln709Arg						
#14	10	F	*BRAF*	p.Gln257Arg		+	+	+	+	
#15	14	F	*BRAF*	p.Trp531Arg	+	+	+	+	+	
#16	15	F	*BRAF*	p.Gln257Arg			+	+	+	
#17	16	M	*BRAF*	p.Thr470Pro		+	+	+	+	
#18	3	M	***BRAF***	**p.Gln257Arg**			+	+	+	**20 months**
#19	10	M	*MAP2K2*	p.Ala62Pro	+	+	+	+	+	
#20	1.5	M	***BRAF***	**p.Lys601Gln**			+	+	+	**1.5**
#21	7	M	*BRAF*	p.Phe468Ser	+		+			
#22	30	F	***KRAS***	**p.Val14Il**			+	+	+	**4**
#23	23	F	*BRAF*	p.Leu485Phe			+			
#24	38	M	***MAP2K1***	**p.Leu42Phe**		+	+	+		**2.5**
#25	18	F	*BRAF*	p.Leu525Pro	+		+		+	
#26	11	F	*BRAF*	p.Gln257Arg	+	+	+	+	+	

+ = presence, G-Tube = gastrostomy tube, pt = patient, y = years. In **bold**, genetic findings linked to long-term worst feeding outcome.

**Table 3:** The I-MCH-FS scores.

Individual Item	Cohort (*n* = 9)	
	Mean ± SD	Median
1 How do you find mealtimes with your child?	3.7 ± 2.1	4.0
2 How worried are you about your child’s eating?	4.2 ± 2.5	5.0
3 How much appetite (hunger) does your child have?	3.0 ± 2.4	1.0
4 When does your child start refusing to eat during mealtimes?	4.3 ± 2.1	4.0
5 How long do mealtimes take for your child (in minutes)?	4.8 ± 1.9	5.0
6 How does your child behave during mealtimes?	5.1 ± 2.0	5.0
7 Does your child gag or spit or vomit with certain types of food?	6.3 ± 2.1	7.0
8 Does your child hold food in his/her mouth without swallowing it?	1.8 ± 1.2	1.0
9 Do you have to follow your child around or use distractions (toys, TV) so that your child will eat?	4.8 ± 2.7	7.0
10 Do you have to force your child to eat or drink?	2.4 ± 1.8	1.0
11 How are your child’s chewing (or sucking) abilities?	2.8 ± 2.2	1.0
12 How do you find your child’s growth?	5.3 ± 2.5	7.0
13 How does your child’s feeding influence your relationship with him/her?	1.3 ± 0.9	1.0
14 How does your child’s feeding influence your family relationships?	6.3 ± 1.2	7.0
Total raw scores	60.3 ± 16.0	61.0
T-score equivalents	64.9 ± 3.2	66.0

## Data Availability

The data generated during the current study are available from the corresponding author upon reasonable request.

## Supplementary Materials

The following supporting information can be downloaded at https://www.mdpi.com/article/10.3390/genes14071338/s1: Table S1: STROBE Statement—Checklist of items that should be included in reports of cross-sectional studies.

## Abbreviations

CFCS	Cardiofaciocutaneous syndrome
CS	Costello syndrome
NS	Noonan syndrome
ASD	Autism spectrum disorder
CP	Cerebral palsy
DQ5	5 min Drooling Quotient
GERD	Gastro oesophageal reflux disease
I-MCH-FS	Italian version of the Montreal Children’s Hospital Feeding Scale
SP	Sensory Profile
